# Discovering Loci for Breeding Prospective and Phenology in Wheat Mediterranean Landraces by Environmental and eigenGWAS

**DOI:** 10.3390/ijms24021700

**Published:** 2023-01-15

**Authors:** Venkata Rami Reddy Yannam, Rubén Rufo, Ilaria Marcotuli, Agata Gadaleta, Marta S. Lopes, Jose Miguel Soriano

**Affiliations:** 1 Sustainable Field Crops Programme, IRTA (Institute for Food and Agricultural Research and Technology), 25198 Lleida, Spain; 2 Department of Soil, Plant and Food Science (Di.S.S.P.A.), University of Bari Aldo Moro, 70126 Bari, Italy

**Keywords:** climate change, association mapping, wheat landraces, synteny, candidate genes

## Abstract

Knowledge of the genetic basis of traits controlling phenology, differentiation patterns, and environmental adaptation is essential to develop new cultivars under climate change conditions. Landrace collections are an appropriate platform to study the hidden variation caused by crop breeding. The use of genome-wide association analysis for phenology, climatic data and differentiation among Mediterranean landraces led to the identification of 651 marker-trait associations that could be grouped in 46 QTL hotspots. A candidate gene analysis using the annotation of the genome sequence of the wheat cultivar ‘Chinese Spring’ detected 1097 gene models within 33 selected QTL hotspots. From all the gene models, 42 were shown to be differentially expressed (upregulated) under abiotic stress conditions, and 9 were selected based on their levels of expression. Different gene families previously reported for their involvement in different stress responses were found (protein kinases, ras-like GTP binding proteins and ethylene-responsive transcription factors). Finally, the synteny analysis in the QTL hotspots regions among the genomes of wheat and other cereal species identified 23, 21 and 7 ortho-QTLs for *Brachypodium*, rice and maize, respectively, confirming the importance of these loci.

## 1. Introduction

Climate change may be the single unifying and chronic issue that will affect everyone and every aspect of the economy. Changes in weather patterns and variability, and differential combinations of effects in different parts of the world, including the Mediterranean region, are expected. The Mediterranean Basin embraces countries between 27° and 47° N and between 10° W and 37° E extending over three continents and a coastline of 46,000 km [1]. Recent accelerated climate change has exacerbated existing environmental problems in the Mediterranean Basin that are caused by the combination of changes in land use, increasing pollution and declining biodiversity. The expected change in the global climate will significantly affect wheat production, with an extraordinary impact in the Mediterranean basin, where prediction models have projected a rise of temperatures by 3–5 °C and a decrease of annual rainfall by 25–30% in the next decades [2].

In the Mediterranean Basin, wheat is mainly cultivated under rainfed conditions with an irregular precipitation pattern across years and locations, and along the plant growth cycle resulting in major yield variations [3]. In addition, wheat usually experiences terminal drought originated by high temperatures during the grain-filling period [4], causing a reduction in yield potential of about 50% [5]. Therefore, there is a need to improve the selection of crops to be able to maintain acceptable levels of yield and stability in semi-arid environments, which have been identified as the regions most sensitive to the effects of climate change [6]. To achieve that, strategies to retain and increase genetic diversity are being explored since climate change is expected to constrain it [7]. The adaptability and stability of new cultivars that can be successfully grown in dry areas will be the main concern in breeding programs.

The ‘green revolution’ based on dwarfing genes, photoperiod insensitivity and high yield potential had a demonstrable impact in breeding. However, it was soon sensed that there was a need to accelerate the use of unique genetic diversity. Future yield gains, especially under stressed conditions, will require the exploitation of the largely untapped sources of genetic diversity housed in collections of wheat landraces and wild relatives [8]. Landraces are genetically diverse repositories of unique traits that have evolved in local environments characterized by a wide range of biotic and abiotic conditions. Landraces were developed during the evolution of wheat along new territories by human selection after the advent of agriculture. Mediterranean landraces have a good adaptation to their environments, forming populations with different genetic constitutions and are the reservoir of the greatest genetic variability of the species [1]. The pioneer Mediterranean farmers started selecting the plants with the most favourable characteristics in terms of vigour, phenological adaptation, spike length, and yield with the aim to produce improved lines [1]. Wheat landrace collections contain wider genetic diversity than most breeding programs, including adaptation to different conditions according to the place of origin [9]. Knowledge of the genetic diversity and population structure of landraces is essential for their conservation and efficient use in breeding programs [10,11], especially concerning the field of adaptation to climate change [9]. To achieve that, several studies using molecular markers such as simple sequence repeats (SSRs) or single nucleotide polymorphism (SNP) are currently conducting since they have been proven to be very useful for evaluating the genetic diversity and population structure of Mediterranean wheat collections [10,12]. Several efforts were carried out to identify the genetic loci responsible for the changes that occurred at the genome level in wheat during the breeding process by eigenGWAS [13,14].

Under drought conditions, wheat productivity can vary depending on the phenological stage at which the water deficit occurs [15], being larger when water is limited at reproductive stages than if it occurs only at the vegetative stage [16]. Therefore, matching phenology to growing season length, changing the cultivar day length and temperature response could be a good prospect of adaptation to climate change [17]. The genetics of flowering time in wheat is complex due to a strong genotype x environment (GxE) interaction [18]. The genetics of wheat development are determined mainly by the allelic diversity within the loci regulating the vernalization requirement (Vrn) and photoperiod sensitivity (Ppd). The third group of genes controlling earliness when the vernalization and photoperiod requirements are accounted for are the earliness per se loci (Eps), characterized by a polygenic inheritance and lower effects than Vrn and Ppd loci [19].

To support breeding for the development of climate-adapted cultivars, different studies have investigated the impact of the environment in domestication and found evidence of swift evolution in response to environmental changes [20]. New approaches using long-term climatic data of the regions of origin of germplasm collections in combination with genomic analyses are useful for detecting genome regions controlling adaptation to environmental conditions. It is the so-called environmental (env)GWAS [14,21].

In the present study, we aimed to identify the genomic regions from a collection of wheat Mediterranean landraces controlling (1) the phenology fitting as a mechanism to escape drought and heat episodes, (2) the response to environmental conditions by envGWAS, and (3) the differentiation patterns among the landrace subpopulations by eigenGWAS.

## 2. Results

### 2.1. Climatic Data and Phenology Assessment

Long-term climatic data of the 23 countries origin of de Mediterranean landraces (Supplementary Materials Table S1) were collected. A period of 15 years was used to determine the following variables: average daily values for minimum, maximum, and mean temperatures (Tmin, Tmax and Tmean, °C), sunshine (h), solar radiation (Rad, MJ m^−2^ day^−1^), relative air humidity (Rh, %), potential evapotranspiration (ET0, mm) and rainfall (Rain, mm). Climatic data from each country were averaged for two main periods: sowing to anthesis (SA) and anthesis to maturity (AM) (Table 1).

Field trials were carried out in a typical Mediterranean climate characterized by an irregular pattern of rainfall distribution during the year, low temperatures in winter that rise abruptly in spring, and high temperatures until the end of the crop cycle. Table S2 shows the rainfall and maximum and minimum temperatures during the crop cycle in the 3 years of field trials and the average of the last 15 years (2006–2021). Precipitation and temperature values were representatives of long-term data from the region for each growing season. However, 2017 was considered exceptionally dry due to the low rainfall. On the other hand, 2018 was the wettest year during the crop cycle, with 262 mm of rainfall (higher than the average of 15 years), whereas the first and second growing seasons, with 207 and 179 mm, respectively, were rather dry. The crop suffered severe water scarcity during the grain filling period in 2016 (3.5 mm) and 2018 (8.7 mm). The years with the lowest rainfall before and after booting were 2016 (22.2 mm) and 2017 (25 mm), respectively. The warmest winter occurred in 2016, especially during January and February, with temperatures above the average for the period of 15 years.

Results of ANOVA showed that all the traits showed statistical differences for the year, climatic zone and genetic subpopulation. In contrast, when the interaction between year and climatic zone, and year and genetic structure were considered, only D87 and GFD were statistically significantly different at *p* < 0.05 (Table 2).

Comparisons of the mean values of the phenology traits recorded in landraces during the three years of field trials (Table S3) revealed significant differences across years for all traits (Table 2). The shortest growing cycle was found during 2016, whereas this year also showed the longest DBA and GFD. In addition, 2017 showed the shortest periods from booting to anthesis and grain filling. Considering the climatic zones defined by [22], a clear separation between the north and south of the Mediterranean basin is reflected. No significant differences were observed between North Balkan and North Coast zones, whereas, for those in the south, only significant differences were observed for DBA. Landraces from the south of the Mediterranean showed shorter crop cycle. According to their genetic structure, although not statistically significant, those landraces from northern Mediterranean countries showed a longer crop cycle but shorter GFD (statistically significant) due to a longer period till anthesis. Western and eastern Mediterranean landraces did not show statistically significant differences in phenology except for DBA with a shorter period for eastern landraces.

**Figure 1:**
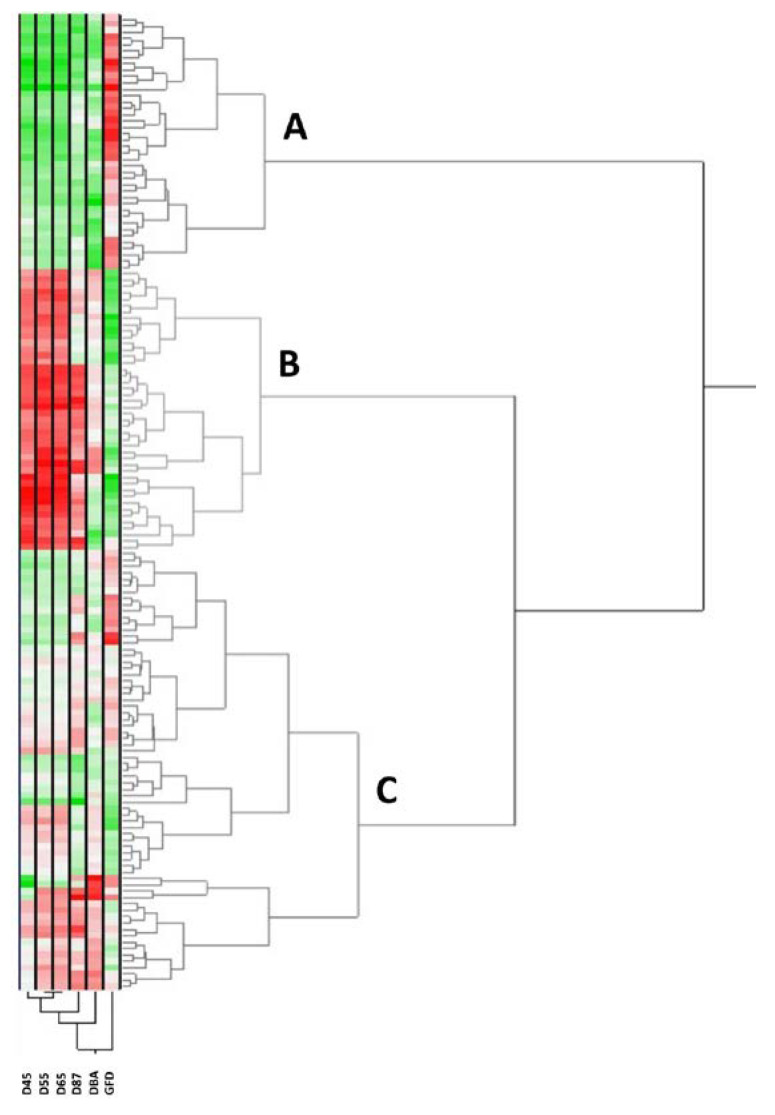
Bidimensional clustering showing the phenotypic relationships between the 153 bread wheat landraces based on the traits specified in the vertical cluster at the bottom. Red and green colours in the columns indicate high and low values, respectively. Dark, higher values; light, lower values; white, intermediate values. D45 (boots swollen), D55 (heading), D65 (anthesis), D87 (physiological maturity), DBA (days from booting to anthesis) and GFD (grain filling duration).

The bidimensional clustering shown in Figure 1 represents the relationships among accessions and their mean phenotypic performances. The horizontal cluster grouped the landraces according to their phenotypic similarity according to the traits in the vertical cluster. The horizontal clustering separated three main groups. Group A was characterised by longer GFD but lower values for the rest of the traits, whereas group B was characterised by shorter GFD but longer D45, D55, D65 and D87. The first cluster within this group is distinguished because of the shorter D87. Group C showed intermediate values, separated into two main clusters based on higher values of the traits except for GFD. Groups A and C are composed mainly of south Mediterranean landraces (76% and 61%, respectively), whereas 95% of the landraces in group B corresponded to north Mediterranean landraces. From the total of southern landraces in group A, 68% corresponded to the South East climatic zone defined by Royo et al. [22], whereas 90% of the southern landraces in group C corresponded to the South West climatic zone. If the genetic subpopulation (SP) is considered, the main SP for the groups A, B and C were SP3 (East Mediterranean) (47%), SP2 (North Mediterranean) (68%), and SP1 (West Mediterranean) (51%), respectively.

### 2.2. Detection of Loci for Phenology Adjustment

A total of 306 MTAs were detected for phenology traits, with D55 showing the highest number of associations (75) and DBA the lowest (32). Seventeen chromosomes reported MTAs (Table S4). Chromosome 7A reported 48 associations, most of them (60%) at 208 cM, followed by chromosome 1B with 42. Chromosomes 1D and 7D harboured only one MTA, and chromosome 3A, only 2.

### 2.3. Detection of Loci for Climatic Variables by envGWAS

Climatic variables were separated into two groups: (1) sowing to anthesis (SA), and (2) anthesis to maturity (AM). Sixty-three MTAs corresponded to the first period and 195 to the second one. The number of MTAs from SA ranged from 11 for Rh and solar radiation to 5 for Tmin and Tmean, whereas AM ranged from 93 for Tmin to 5 for rainfall (Table S4). The MTAs for climatic variables were detected throughout the genome except for 2D and 4D chromosomes. Chromosome 1D reported the maximum number of MTAs (75), and all of them were related to Tmin during the AM period and were in a region of approximately 19 cM (160.85–179.54). On the other hand, chromosomes 3D and 7D reported the lowest number of MTAs (2).

### 2.4. Detection of Loci for Genetic Differentiation by eigenGWAS

After data filtering (duplicated patterns, markers with more than 25% of missing values, genotypes and markers with minor allele frequency lower than 5%), a total of 10,458 SNPs were used for molecular analyses of the bread wheat Mediterranean landraces, as reported by Rufo et al. [12].

**Figure 2:**
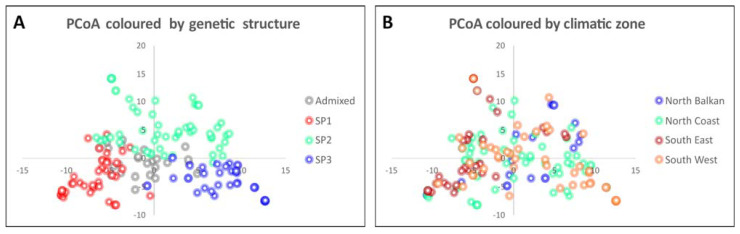
PCoA with a collection of 153 bread wheat Mediterranean landraces based on 10,458 SNPs. The genotypes are coloured based on: (**A**) the genetic structure [12] and (**B**) the climatic zone of the country of origin as defined by Royo et al. [22].

EigenGWAS was conducted using the top five eigenvectors resulting from the PCoA obtained for the whole collection of genotypes (Figure 2). Genotypes were clearly separated by their genetic SP [12] into three clusters based on their origin: West Mediterranean (SP1), North Mediterranean (SP2), and East Mediterranean (SP3). Genotypes with a high level of admixture remained in a central position between the three SPs (Figure 1A). A high level of admixture was observed when genotypes are identified by their climatic zone (Figure 1B). South East genotypes were mostly clustered on the negative axes, opposite to the north Balkan genotypes. In comparison, those from the North Coast and South West (the regions with intermediate values) were widely distributed in the PCoA.

The largest eigenvalue was 4075.3, explaining 7% of the genetic variation, whereas the 5th eigenvalue was 1601.6, explaining 3% of the genetic variation. The top five eigenvalues accounted for 22% of the genetic variation, which indicates the complexity of the population structure of the collection. 

A total of 87 MTAs were identified for the top five eigenvectors using a moderate threshold of -log10 *p* = 3.0 (Table S4) in 16 out of the 21 wheat chromosomes. Number of MTAs ranged from 15 in chromosome 7A to 1 in chromosome 4A. The mean percentage of variance explained (r2) per MTA ranged from 0.001 to 0.102, with an average of 0.020.

### 2.5. Identification of QTL Hotspots

**Figure 3:**
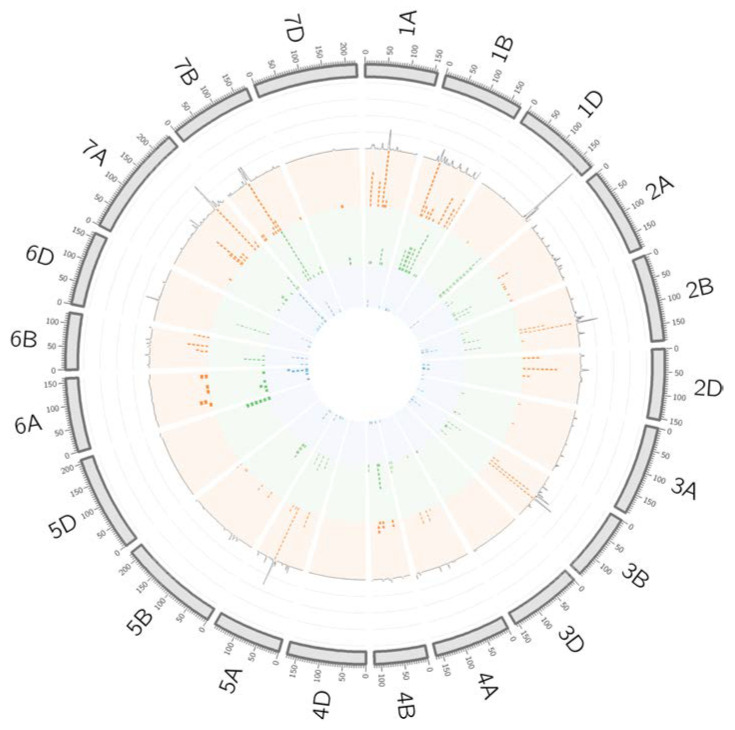
QTL overview index. The index values are represented along chromosomes as a grey line. Bars below the QTL overview index represent the significant MTAs (−log_10_ *p* > 3). Orange, phenology MTAs; green, climatic MTAs; blue, eigen MTAs.

To simplify all this information and to identify consensus genomic regions controlling different traits, QTL hotspots were identified using the QTL overview index defined by Chardon et al. [23] for each cM of the genetic map reported by Wang et al. [24]. Confidence intervals (CIs) were calculated based on the extent of the linkage disequilibrium (LD) for each chromosome following Rufo et al. [12]. A total of 567 positions were detected using as a threshold the mean of the overview index across the 21 chromosomes (0.19), corresponding to 153 peaks. In contrast, using a high threshold (0.93), a total of 173 positions were identified over the threshold, corresponding to 69 peaks (Figure 3).

These 69 peaks were reduced to 46 QTL hotspots (Table S5), 16 in genome A, 26 in genome B and 4 in genome D. To simplify the search for candidate genes (CGs), QTL hotspots were excluded when the CI was higher than 20 Mb (Table 3).

Thirty-three QTL hotspots remained for subsequent analyses, with 12, 18 and 3 hotspots for genomes A, B and D, respectively. When the physical position of the SNPs was considered, QTL hotspots 1B.1 and 1B.2, and 1D.1 and 1D.2 showed overlapping positions, thus, they were considered single hotspots (1B.1–2 and 1D.1–2). According to the physical positions for functional genes reported by Liu et al. [25], some of the QTL hotspots co-localize with them. Sec-1, linked to the 1B/1R translocation, was found to be within the hotspot 1B.3, the flowering genes TaELF3-B1 and TaELF3-D1 corresponded to the hotspots 1B.8 and 1D.2, respectively, the photoperiod sensitivity gene Ppd-A1 was in hotspot 2A.1 and the vernalization gene Vrn-A1 in 5A.3. Finally, the position of the phytoene synthase gene Psy-A1 matched with the position of QTL hotspot 7A.5.

To identify the genomic regions most involved in subpopulation differentiation, QTL hotspots detected by eigenGWAS were analysed for allelic frequencies within them. Eight out of the thirteen QTL hotspots showed differences in the allele frequency of the markers associated with differentiation patterns (Table 4). A threshold of allele frequency within a group was set at 80% to identify robust differences among groups, whereas moderate differences were determined at 60%. The most considerable difference among SPs was for QTL hotspot 7B.2, where SP1 showed a clear different pattern from the other SPs. QTL hotspot 2B.1 helped to distinguish SP2 from SP3 with a moderate and robust threshold, respectively. A moderate threshold was observed in QTL hotspot 5B.1 to differentiate SP3 from the other SPs. The marker RAC875_c19099_434 (T/C) in QTL hotspot 5B.3 discriminates SP1 (T allele) from SP3 (C allele), whereas GENE-1074_108 (T/C) in QTL hotspot 6B.2, SP1 (T allele) from SP2 (C allele).

### 2.6. In Silico Analysis of Candidate Genes

A total of 1097 candidate genes (CG) were found within the selected 33 QTL hotspots (Table S6). A total of 371 gene families were observed among the CGs, 10% of the genes corresponded to receptor-like kinases, 6% to F-box proteins and 4% to disease resistance proteins.

To classify these genes according to their molecular function (MF), biological process (BP) and cellular component (CC) gene ontology (GO) terms were downloaded from https://wheaturgi.versailles.inra.fr/Seq-Repository/Annotations (accessed on 1 September 2022). Eight hundred and seven CGs were classified according to their MF, 148 according to their BP and 78 according to their CC. The main gene families within MF ontology were protein binding (26% of the genes), nucleic acid binding (16%) and protein kinase activity (13%). For their BP, genes involved in oxidation–reduction processes were 23%, followed by metabolic process (21%) and proteolysis (9%). Finally, according to their cellular location, 45%, 17% and 13% of the genes corresponded to membrane, ribosome, and nucleosome, respectively.

**Figure 4:**
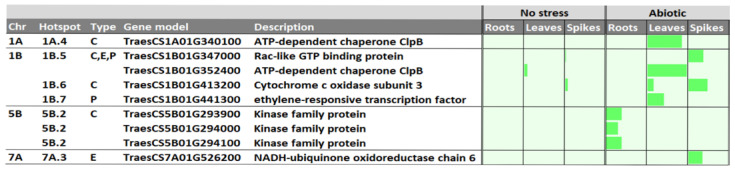
Upregulated CGs under abiotic stress conditions in three tissues Values are based on log2 tpm (dark green). CGs, candidate genes; tpm, transcripts per million. The length of the cells corresponds to 7.2 tpm.

Subsequently, a search for differentially expressed genes (DEGs) upregulated under three abiotic stress conditions reported in http://www.wheat-expression.com (accessed on 1 September 2022) was conducted to identify the best CGs. These conditions comprised (1) drought and heat stress time-course in seedlings, (2) spikes with water stress, and (3) seedlings treated with polyethylene glycol (PEG) to simulate drought. Forty-two CGs were filtered by performing DEG in different tissues: roots, shoots/leaves, spikes, and grain. To reduce the complexity of the analysis, only those DEGs with a difference larger than 2 tpm from no stress to abiotic stress conditions were considered, leaving 9 CGs that were upregulated under abiotic stress in 8 QTL hotspots from 4 chromosomes (Figure 4). From them, 3 DEG corresponded to receptor-like kinases expressed in roots under drought stress. Two transcripts were ATP-dependent chaperone ClpB upregulated in shoots/leaves. The other gene families corresponded to an ethylene-responsive transcription factor, also upregulated in shoots/leaves; a cytochrome c oxidase subunit 3 upregulated in shoots/leaves and spikes, and, finally, a Ras-like GTP binding protein and NADH-ubiquinone oxidoreductase chain 6 upregulated in the spikes. Any of the genes were found to be upregulated in the grain. Four DEGs were located on chromosome 1B 3 on 5B, and one on chromosomes 1A and 7A.

### 2.7. Synteny Analysis within Cereal Species

**Figure 5:**
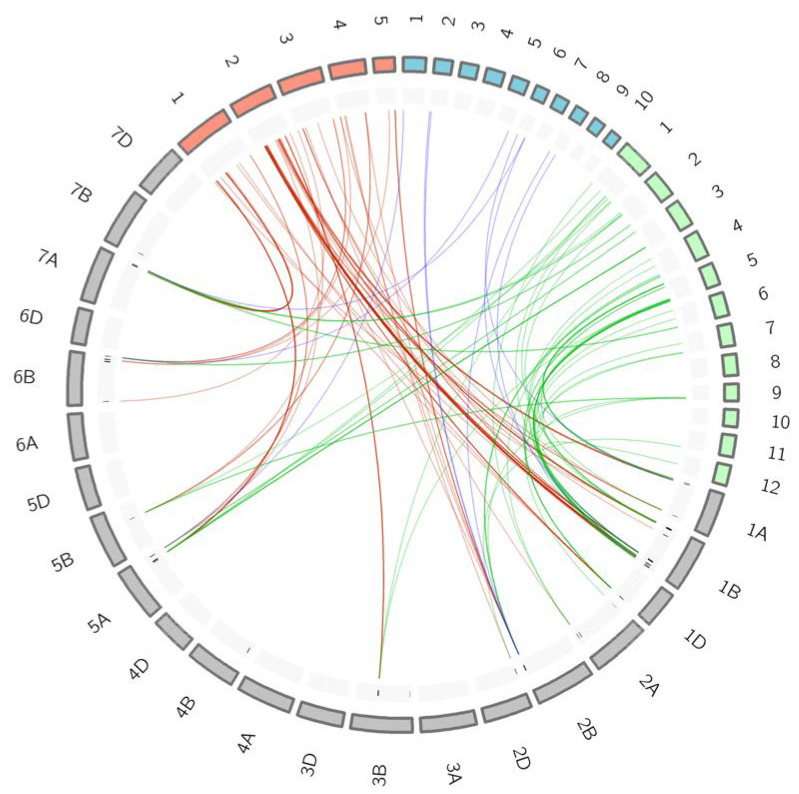
Synteny among genomes for the selected QTL hotspots. For better visualization of the chromosome links, the size of the *Brachypodium* and rice chromosomes is multiplied by 10. Colour code: red, *Brachypodium*; blue, maize; green, rice; grey, wheat.

Markers from the selected QTL hotspots were launched against the mapping pipeline of the Brachypodium, rice and maize genomes, as reported in Marcotuli et al. [26]. Twenty-four out of the 33 selected QTL hotspots reported collinearity with the other genomes. The number of collinear QTL hotspots was 23, 21 and 7 for Brachypodium, rice and maize, respectively. From those hotspots, a total of 97 markers were identified in 79 syntenic regions of the three genomes, designated as ortho-QTL (Table 5). From them, 84, 57, and 9 markers were common with Brachypodium, rice and maize genomes. Among those genomes, 40 markers were in common between Brachypodium and rice, 3 between Brachypodium and maize, and 7 markers were in common in the three genomes (Figure 5).

Examining collinear regions within the four genomes outcomes in identifying 20 ortho-QTL containing 41 orthologous genes with similar functions that can be considered promising candidate genes controlling traits of interest across species. From them, 27 genes were orthologous between wheat and Brachypodium, 4 between wheat and rice, 2 between wheat and maize, 5 between wheat, Brachypodium and rice, 2 between wheat, Brachypodium and maize, and, finally, 1 gene was orthologous in the four species (Table S7).

## 3. Discussion

Genetic diversity is crucial in plant breeding as it broadens the source of new alleles for essential genes. The use of wild relatives or landraces that are well adapted to their regions of origin is of special interest when breeding in suboptimal environments such as the Mediterranean basin to improve the modern cultivars to face the challenges of climate change [19]. In a previous study using the same collection of landraces, Royo et al. [22] found differences in the agronomic performance of the landraces from the northern and southern areas of the Mediterranean Sea. The authors found a clear association between these traits and the climatic conditions of the countries of origin of the landraces. According to these results in this study, we aimed to identify the genomic regions from the Mediterranean wheat landraces controlling (1) the phenology fitting as a mechanism to escape drought episodes, (2) the response to environmental conditions by envGWAS, and (3) the differentiation patterns among the landrace subpopulations by eigenGWAS.

### 3.1. Phenology Fitting

Phenology was differentiated by climatic zone, increasing from the warmest and driest regions to the coldest and wettest ones, showing statistically significant differences between the north and south coast but not within them. This agrees with Gooding et al. [27], who reported a reduction in flowering time in wheat in high temperature and drought environments during the crop cycle. When the phenology is compared by the population structure, SP2 showed a longer cycle till booting, heading and anthesis, but shorter GFD; although this is contradictory according to the geographical distribution of SP2 landraces, mainly north Mediterranean countries, it may be explained by the higher level of admixture among Mediterranean landraces SP as reported by Rufo et al. [12]. However, other studies [28] reported a decrease in GFD due to late anthesis. The bidimensional clustering was helpful to visualize the variability within the collection and identifying phenological connections among landraces. The classification followed mainly the separation among climatic zones into three groups, South West (group A), north Mediterranean (group B, including north coast and north Balkan) and South East (group C). However, identifying a trend to distinguish genetic subpopulations according to phenology data was not as good as with climatic zones.

The dissection of genetic architecture for phenology in Mediterranean wheat landraces revealed the presence of 26 QTL hotspots (57%) with significant associations. According to the position of functional genes described by Liu et al. [29], major genes affecting photoperiod sensitivity and vernalization were in QTL hotspots 2A.1 (*Ppd-A1*) and 5A.3 (*Vrn-A1*), respectively. However, no MTAs were found in the homologous regions corresponding to the *Ppd* and *Vrn genes* on chromosomes 2B and 2D, and 5B and 5D, respectively. These contrast regions that were detected might be due to selection pressure during the domestication by selecting the alleles/genes targeted to environmental factors, growing type, phenological traits, yield and local adaptation. Continuity of the research in these regions can lead to underpinning the genes controlling these loci. Dissecting the complex genetic architecture to identify the favorable regions and haplotypes could be beneficial for the development of new varieties able to skip the drought periods by regulating their phenological stages.

### 3.2. Genetic Control of Environmental Conditions

EnvGWAS has been reported as a valuable and complementary approach to identify genomic regions related to adaptation to abiotic stress [21]. The hierarchical clustering reported by Royo et al. [22] using the long-term climatic data of the main growing areas of wheat in the Mediterranean basin showed a clear geographic pattern separating the north coast countries from the southern ones. Climatic data are highly correlated with the adaptation of the crop. As it is shown by different authors [22,29], PCoA using molecular data followed the climatic classification reported by these authors when genotypes were grouped by their country of origin. However, the high level of admixture and genetic exchange in the Mediterranean landraces according to Rufo et al. [12] revealed an unclear pattern when comparing PCoA using structure or climatic data.

In the present work, envGWAS, using the climatic variables from the region of origin of the Mediterranean wheat landraces, successfully detected genome regions involved in the control of traits related to environmental variation. QTL hotspots showed that most of the significant associations related to temperature were reported during the grain filling period, which, according to Royo et al. [22], is one of the variables that most contribute to differentiate the landraces from the north and south coast of the Mediterranean basin. Taking advantage of the benefits of envGWAS can lead to improving the knowledge of the adaptation of the Mediterranean landraces to their specific environments.

### 3.3. Genetic Loci for Differentiation Patterns among Mediterranean Landraces

Eigenvectors are commonly used to deduce the population’s genetic structure because they are estimated for each genotype. In this way, different studies have indicated the suitability of primary eigenvectors to infer population differentiation [30,31,32], and posteriorly Chen et al. [33] developed the approach called eigenGWAS to identify genomic regions causing genetic differentiation. This approach has been used mainly to identify selective sweeps produced by breeding among old cultivars or landraces and modern varieties [13,25,34,35]. These authors found that most of the selective sweeps corresponded to regions involved in yield potential, phenology, plant height, and biotic and abiotic resistance.

In this study, we used eigenvectors to understand the genetic differences among wheat Mediterranean landraces. Out of the eleven QTL hotspots reporting significant associations by eigenGWAS, six hotspots on chromosomes 2B, 5B, 6B, 7A and 7B showed allelic or haplotype differences among genetic subpopulations, thus considering them as the main drivers of genetic differentiation among Mediterranean landraces. Four of these regions also showed associations with climate traits and three with phenology fitting, indicating not only genetic differentiation but also adaptive differentiation among different environments. These loci can be beneficial for breeding purposes by spotlighting the breeders to know the contribution of the genomic footprints in the wheat adaptation and response to the climatic change by selecting the high frequency of favorable alleles landraces and integrating them in the breeding programs.

### 3.4. Candidate Genes

To reduce genomic complexity and the number of CGs, the search was performed only in the selected QTL hotspots, such as those hotspots with a confidence interval lower than 20 Mb. The gene annotation from the “Chinese spring” reference genome sequence [36] allowed us to identify 1097 gene models within the 33 QTL hotspots. Mining of CGs was achieved by looking for differentially expressed genes (DEGs) upregulated under abiotic stress in different tissues through in silico analysis at http://www.wheat-expression.com (accessed on 1 September 2022). Only those genes with higher differences between non-stressed conditions and abiotic stress were selected. Among them, different gene families previously reported for their involvement in various stress responses were found: Kinases, Ras-like GTP binding proteins and ethylene responsive transcription factors.

Protein kinases play crucial roles in plant responses to different stress conditions (reviewed in [37]). Among them, the MAPKs are involved in ABA signalling and drought stress regulating root growth and stomatal closure; CPKs play roles in signalling modules in different abiotic stresses such as drought, heat, cold and salt; RLKs are a large group of kinases anchored to the cellular membrane, thus acting in the transmission of extracellular signals into the cells. They regulate fertilization, plant growth and development, and plant response to biotic and abiotic stresses. Differentially expressed kinases were also found in a previous study of QTL meta-analysis for abiotic stress (among others) in durum wheat by Soriano et al. [38].

A Ras-related GTP binding protein was found in hotspot 1B.5. These proteins have been involved in drought stress tolerance by modulating stomatal movements mediated by ABA [39].

Finally, an ethylene-responsive transcription factors (ERF) was upregulated in leaves. These genes have been reported to be involved in different processes. Djmal and Khoudi [40] studied the tolerance to heavy metals (HM) regulated by the durum wheat TdSHN1 in yeast and transgenic tobacco, concluding that the gene could improve HM tolerance in plants and phytoremediation of HM-contaminated soils. Gao et al. [41] studied the expression of the wheat ERF *TaERFL1a*. This gene is a member of the AP2/ERF family and was remarkably induced in wheat seedlings suffering freezing stress. In their study, the authors showed that its expression was rapidly upregulated in response to salt, cold, and water deficiency, suggesting roles in the responses to abiotic stresses.

Interestingly, in QTL hotspot 1B.6, a Cytochrome c oxidase gene was upregulated in leaves and spikes (TraesCS1B01G413200). This gene was previously found to be upregulated in spikes by Rufo et al. [42] in a GWAS for yield and vegetation indices-related traits using the same panel of landraces but also including a panel of modern wheat cultivars.

### 3.5. Synteny among Cereal Species

The study of synteny among different species and the identification of orthologous genes suggest that they may be associated with regulatory elements affecting many genes [43] and leading to the identification of key genes controlling important traits across species. In this study, we used the selected QTL hotspots to investigate the collinearity with other cereal species: *Brachypodium*, maize and rice (ortho-QTL).

The analysis revealed a higher synteny among the wheat, *Brachypodium* and rice, whereas a lower one among wheat and maize. The candidate gene investigation allowed the correlation between some ortho-QTL with genes. Only one gene, a 4-Coumarate: CoA ligase, was common among the four genomes. These proteins were observed to play a pivotal role in cell wall synthesis and abiotic stress in *Fraxinus mandshurica* [44]. The authors found that *Fm4CL* is involved in secondary cell wall development and lignin synthesis. *Fm4CL* plays an important role in osmotic stress by affecting cell wall and stomatal development. In rice, these proteins were involved in fungal resistance induced by reactive oxygen species [45]. The collinearity among cereal genomes could suggest a common mechanism of action to respond at stress conditions, which may facilitate the study of such complex traits.

## 4. Materials and Methods

### 4.1. Plant Material

A panel of 153 bread wheat (*Triticum aestivum* L.) landraces from 23 Mediterranean countries, including Balkan peninsula and Portugal, were used in the current study (Table S1). Landrace populations were provided by public gene banks from Germany (IPK, Gatersleben), Italy (ISC, S. Angelo Lodigiano), Romania (Suceava GenBank, Suceava), Russia (VIR, St. Petersburg), Spain (CRF-INIA, Madrid), the Netherlands (CGN-WUR, Wageningen) and the USA (NSGC-USDA, Aberdeen, ID). Accessions were bulk purified during two cropping cycles to select the dominant type, and the seeds were increased on plots in the same field during 2015 to ensure a common origin for all lines.

### 4.2. Phenology Assessment

Field experiments were conducted under rainfed conditions in Gimenells, Lleida, northeast Spain, during 2016, 2017 and 2018 harvesting seasons, as reported in Rufo et al. [42]. The experiments consisted of a non-replicated augmented design with two replicated checks, the cultivars ‘Anza’ and ‘Soissons’, at a ratio of 1:5 between checks and tested genotypes. The sowing density was adjusted to 250 germinable seeds/m^2^, and the sowing dates were 2 December 2015; 21 November 2016; and 15 November 2017, whereas harvesting dates were 7 July 2016; 5 July 2017; and 5 July 2018. Weeds and diseases were controlled following standard practices at the site.

The development of plants at each plot was monitored twice a week for the following growth stages: D45 (boots swollen), D55 (heading), D65 (anthesis) and D87 (physiological maturity) [46]. A plot was considered to reach a given developmental stage when approximately 50% of the plants presented the specific characteristics of the stage. Days from booting to anthesis (DBA) were calculated as the number of days from booting to anthesis, and grain filling duration (GFD) was calculated as the number of days from anthesis to physiological maturity. 

Phenotypic data were fitted to a linear mixed model considering the check cultivars as the fixed effect, and the row and column number and accessions as random in the model for each environment following the MIXED procedure of the SAS-STAT statistical package (SAS Institute Inc., Cary, NC, USA)

y = Xβ + Zγ + ε (1)

where β is an unknown vector of fixed-effects parameters with known design matrix X, γ is an unknown vector of random-effects parameters with known design matrix Z, and ε is an unknown random error vector whose elements are no longer required to be independent and homogeneous. Restricted maximum likelihood (REML) was used to produce the best linear unbiased predictors (BLUPs).

Analyses of variance (ANOVA) were conducted to assess differences among years and climatic zone or genetic subpopulations. Genotype within a climatic zone or genetic subpopulation was used as an error term. Mean comparisons between years, climatic zone and genetic subpopulation, were performed using the Tukey’s HSD test at *p* ≤ 0.05. Mean phenotypic values across the 3 years were used to perform a hierarchical cluster analysis by the Ward method [47]. Both analyses were carried out using JMP v14.2.0 statistical package (SAS Institute, Inc., Cary, NC, USA).

### 4.3. Environmental Variables

Long-term climatic data of the 23 countries origin of landraces were collected using the CROPWAT software (http://www.fao.org/land-water/databases-and-software/cropwat (accessed on 1 September 2022)) from the CLIMWAT 2.0 FAO database. A period of 15 years of data (2006–2021) from 3 to 7 climatic stations located in the main wheat-growing areas of each country was used to determine the following variables: average daily values for minimum, maximum, and mean temperatures (Tmin, Tmax and Tmean, °C), sunshine (h), solar radiation (Rad, MJ m^−2^ day^−1^), relative air humidity (Rh, %), potential evapotranspiration (ET_0_, mm) and rainfall (Rain, mm). Climatic data from each country were averaged for the periods 20 November–31 March and 1 April–30 June (Table 1), assuming they represent the two main growing periods of wheat in the region; this is from sowing to anthesis (SA) and from anthesis to physiological maturity (AM) as described in Royo et al. [48].

### 4.4. Genome-Wide Association Analyses

Accessions were genotyped with 13177 SNPs from the Illumina Infinium 15K Wheat SNP Chip at Trait Genetics GmbH (Gatersleben, Germany). After marker filtering, 10,458 SNPs were used for subsequent analyses as reported in Rufo et al. [12].

Genome-wide association (GWAS) was performed using Tassel 5.0 software [49] for phenology, environmental variables and the first five eigenvectors. A mixed linear model (MLM) was fitted using a principal component analysis (PCA) matrix with 6 principal components as the fixed effect and a kinship (k) matrix as the random effect (PCA + K model) at the optimum compression level based on the groups defined by the kinship matrix. Compression levels range from “no compression” (compression = 1) when each genotype belongs to its own group, to “maximum compression” (compression = *n*) when all genotypes belong to the same group. A common threshold was established at –log_10_ *p* > 3, as previously reported in the literature [42,50,51,52,53]. Confidence intervals (CIs) for marker–trait associations (MTA) were estimated for each chromosome based on the LD decay reported by Rufo et al. [12] and standardized using the formula reported by Chardon et al. [23]:

(2) $$S_{i}^{2}={\left(\frac{\mathrm{CI}}{3.92}\right)}^{2}$$

where CI is the LD decay for each chromosome. To simplify the MTA information, the associations were grouped into QTL hotspots. To define a hotspot, the density of MTAs along the chromosome was calculated as the QTL overview index [23] for each cM of the genetic map reported by Wang et al. [24]:

(3) $$U=\frac{\frac{nbQTL}{nbE}}{Total\;length\;of\;map}$$

where nbQTL is the number of QTLs and nbE is the total number of experiments.

### 4.5. In Silico Gene Expression Analyses and Synteny against Cereal Genomes

Gene annotation in the QTL hotspots was performed using the gene models for high-confidence genes reported for the wheat genome sequence [36], available at https://wheat-urgi.versailles.inra.fr/Seq-Repository/Annotations (accessed on 1 September 2022). In silico expression analysis and the identification of upregulated gene models were carried out using the RNA-seq data available at http://www.wheat-expression.com/ (accessed on 1 September 2022) [54] for the following studies: (1) drought and heat stress time-course in seedlings, (2) spikes with water stress, and (3) seedlings with PEG to simulate drought. Gene Ontology (GO) data were retrieved from the high-confidence gene annotation at https://wheat-urgi.versailles.inra.fr/Seq-Repository/Annotations (accessed on 1 September 2022).

Search for syntenic regions in *Brachypodium distachyon*, *Oryza sativa* and *Zea mays* was performed as reported in Marcotuli et al. [26]. The following reference genomes were used: *Brachypodium distachyon* version 1 (http://www.plantgdb.org/BdGDB, accessed on 1 September 2022); *Oryza sativa* Japonica Group version IRGSP-1.0 (http://rice.uga.edu, accessed on 1 September 2022), *Zea mays* version AGPv3 (https://www.maizegdb.org, accessed on 1 September 2022).

Circular figures of the QTL hotspots and chromosome synteny were created using the online software Clico FS’ [55], available at http://clicofs.codoncloud.com (accessed on 20 September 2022).

## Figures and Tables

**Table 1:** Mean of the climatic data of a 15-year period (2006–2021) in the countries of origin of the Mediterranean landraces.

Country	N	Tmax	Tmin	Tmean	Rh	Sunshine	Rad	ET_0_	Rain	Tmax	Tmin	Tmean	Rh	Sunshine	Rad	ET_0_	Rain
		Sowing—Anthesis (SA)								Anthesis—Maturity (AM)							
Albania	6	11.8	3.3	7.6	75.7	2.8	7.1	6.0	663.4	23.1	11.6	17.4	63.1	7.2	19.5	11.2	192.2
Algeria	26	16.1	6.1	11.1	75.4	5.4	11.0	200.0	193.4	26.1	13.6	19.9	60.7	8.6	22.1	385.2	78.2
Bosnia & Herzegovina	6	8.8	0.6	4.7	76.0	1.8	5.7	4.9	554.5	21.5	9.7	15.6	64.2	5.4	16.7	10.0	289.8
Bulgaria	6	7.5	0.5	4.0	81.7	3.0	6.9	111.2	180.8	20.6	11.0	15.8	74.0	7.2	19.3	290.3	162.8
Croatia	6	10.1	3.0	6.5	71.6	2.3	6.0	149.0	397.7	21.7	12.0	16.8	63.6	6.1	17.7	292.7	238.3
Cyprus	10	18.2	6.9	12.5	69.7	5.7	11.4	213.5	255.3	27.4	13.0	20.2	55.8	9.5	23.5	411.6	27.3
Egypt	14	21.9	9.8	15.8	67.5	7.1	14.3	333.7	30.6	31.0	16.5	23.8	49.9	9.9	24.4	541.0	2.2
France	24	11.4	3.5	7.4	78.3	3.3	6.9	162.0	255.9	20.8	10.4	15.6	66.8	7.1	18.9	335.6	161.8
Greece	12	14.4	6.0	10.2	74.2	3.2	8.0	196.1	307.8	24.0	13.4	18.7	64.0	6.1	16.5	313.8	82.5
Israel	10	18.7	8.4	13.5	72.0	6.8	13.2	292.1	442.9	27.8	14.1	20.9	56.2	10.9	25.7	542.4	31.2
Italy	24	14.0	6.8	10.4	75.7	3.6	7.9	180.2	282.7	22.5	13.2	17.9	68.6	7.8	20.3	335.6	104.2
Jordan	8	19.6	7.8	13.7	58.9	6.1	12.7	332.3	165.4	30.3	14.7	22.5	45.1	9.1	23.1	535.8	13.5
Lebanon	6	17.9	10.1	14.0	67.0	4.3	10.1	292.0	722.7	23.9	15.4	19.6	66.1	8.4	21.9	404.1	79.0
Libya	6	19.6	8.8	14.2	65.4	6.1	12.6	324.9	123.2	27.8	15.4	21.6	59.8	8.7	22.5	456.3	14.3
Macedonia	8	8.2	−0.9	3.6	81.3	2.1	6.4	100.2	228.2	22.5	9.0	15.7	64.7	6.3	18.3	296.0	142.1
Morocco	40	18.9	7.2	13.0	69.8	5.9	12.1	262.7	227.6	25.1	12.2	18.6	66.1	8.7	22.4	364.9	84.0
Portugal	8	11.7	3.7	7.7	77.3	4.7	9.1	174.8	630.4	19.6	8.5	14.1	63.6	8.5	21.4	343.3	227.7
Romania	8	6.4	−1.0	2.7	88.7	3.1	6.5	3.6	200.0	21.5	10.4	15.9	72.8	7.8	19.8	10.8	170.0
Serbia	8	7.7	−0.1	3.8	79.7	1.8	5.8	96.0	215.2	21.8	10.3	16.1	65.7	5.7	17.2	278.1	216.0
Spain	22	13.0	4.0	8.5	77.5	3.9	8.4	163.3	226.4	23.0	10.5	16.8	58.5	7.8	20.4	357.9	119.0
Syria	22	15.4	4.4	9.9	71.7	5.1	10.6	212.8	178.8	29.7	14.7	22.2	44.1	9.2	23.0	580.2	38.8
Tunisia	10	17.9	8.3	13.1	72.7	5.6	11.1	244.6	182.5	26.0	14.7	20.3	67.5	8.5	21.9	393.9	45.6
Turkey	30	13.0	4.5	8.8	69.4	3.8	8.6	172.7	400.9	24.3	13.2	18.7	61.6	8.0	20.9	356.0	131.6

Tmax, maximum temperature (°C); Tmin, mínimum temperature (°C); Tmean, mean temperature (°C); Rh, relative humidity (%); Sunshine (h); Rad, radiation (MJ m^−2^ day^−1^); ET, evapo-transpiration (mm); Rain, rainfall (mm)

**Table 2:** Means and ANOVA *p*-values for the phenology traits analysed in the panel of 153 Mediterranean bread wheat landraces included in the study. Data for each climatic zone and subpopulation (SP) represent the mean values across the 3 years. Different letters at each growing period indicate significant differences at *p* ≤ 0.05 using the Tukey’s test.

	Year (Y)				Climatic Zone (CZ)					Genetic Structure (GS)				ANOVA Interactions	
	2016	2017	2018	ANOVA	NB	NC	SW	SE	ANOVA	SP1	SP2	SP3	ANOVA	Y x CZ	Y x GS
D45	136.3 ^a^	145.0 ^b^	157.5 ^c^	<0.0001	150.2 ^a^	147.7 ^a^	144.8 ^b^	143.0 ^b^	<0.0001	144.8 ^b^	148.4 ^a^	146.2 ^ab^	<0.0001	0.8669	0.8202
D55	145.6 ^a^	153.8 ^b^	167.1 ^c^	<0.0001	160.3 ^a^	157.7 ^a^	153.8 ^b^	150.3 ^b^	<0.0001	153.9 ^b^	158.4 ^a^	154.3 ^b^	<0.0001	0.9846	0.9617
D65	151.8 ^a^	158.7 ^b^	172.2 ^c^	<0.0001	165.2 ^a^	162.7 ^a^	159.4 ^b^	156.6 ^b^	<0.0001	159.5 ^b^	163.4 ^a^	159.9 ^b^	<0.0001	0.3626	0.5910
D87	186.0 ^a^	187.8 ^b^	204.3 ^c^	<0.0001	195.0 ^a^	194.0 ^a^	192.1 ^ab^	190.7 ^b^	<0.0001	192.2 ^a^	194.3 ^a^	192.1 ^a^	<0.0001	<0.0001	0.0034
DBA	15.5 ^a^	13.4 ^b^	14.5 ^c^	<0.0001	15.1 ^a^	14.8 ^a^	14.6 ^a^	13.6 ^b^	0.0001	14.6 ^a^	14.9 ^a^	13.8 ^b^	0.0001	0.1049	0.2895
GFD	34.0 ^a^	29.1 ^b^	32.1 ^c^	<0.0001	34.0 ^a^	32.4 ^a^	30.7 ^b^	29.8 ^b^	<0.0001	32.3 ^a^	30.5 ^b^	32.0 ^a^	<0.0001	<0.0001	<0.0001

D45, days from sowing to boots swollen; D55, days from sowing to heading; D65, days from sowing to anthesis; D87, days from sowing to physiological maturity; DBA, days from booting to anthesis; GFD grain filling duration; NB, North Balkan; NC, North Coast; SW, South West; SE, South East. SP1, Western Mediterranean landraces; SP2, Northern Mediterranean landraces; SP3, Eastern Mediterranean landraces.

**Table 3:** Selected QTL hotspots. Chr, chromosome; C, climatic trait; E, eigen vectors; P, phenology trait.

Hotspot	Chr	CI (cM)	Trait	Left Marker	Position (bp)	Right Marker	Position (bp)	CI (Mb)	N Gene Models
hotspot 1A.1	1A	21–27	C,E,P	BS00023201_51	7643102	Excalibur_c71158_54	8296998	0.65	19
hotspot 1A.2	1A	51–53	P	Excalibur_c10689_254	27363007	Kukri_c22508_119	28757949	1.39	9
hotspot 1A.4	1A	95–96	C	BS00062876_51	529788778	BobWhite_c96_170	531682571	1.89	34
hotspot 1B.1	1B	43–45	C	BS00065053_51	38833829	wsnp_Ex_c5780_10153638	26186242	12.65	89
hotspot 1B.2	1B	51–53	P	Excalibur_c95656_129	44933589	Tdurum_contig56188_569	28563880	16.37	114
hotspot 1B.3	1B	62–64	C	wsnp_BE399980B_Ta_2_1	142523393	BS00003575_51	148898343	6.37	27
hotspot 1B.5	1B	86–91	C,E,P	Kukri_c25961_166	575863858	BobWhite_c39656_106	589919646	14.06	147
hotspot 1B.6	1B	111–116	C	BS00094237_51	638015155	BS00084895_51	643101677	5.09	78
hotspot 1B.7	1B	1345–136	P	GENE-0063_68	661515587	Excalibur_rep_c71107_517	664599715	3.08	35
hotspot 1B.8	1B	159–161	P	GENE-0543_201	681690469	Excalibur_rep_c69522_83	685865389	4.17	41
hotspot 1B.9	1B	172–174	P	wsnp_Ex_c1597_3045682	688283056	wsnp_Ku_c13952_22097856	687413792	0.87	9
hotspot 1D.1	1D	161–172	C	RAC875_c14613_68	485557589	RFL_Contig3395_1575	487168787	1.61	34
hotspot 1D.2	1D	179–180	C	BS00093275_51	486241852	Tdurum_contig29915_167	491043383	4.80	75
hotspot 2A.1	2A	74–75	P	Tdurum_contig11803_306	36041083	Ku_c269_2643	36632073	0.59	15
hotspot 2A.2	2A	122–123	C	BS00107804_51	707040172	wsnp_Ex_rep_c66358_64543401	709701422	2.66	30
hotspot 2A.3	2A	143–144	C	BS00062732_51	747090405	Excalibur_c18514_238	750595232	3.50	90
hotspot 2B.3	2B	147–148	C	BobWhite_c12911_788	780590397	BS00100118_51	788524935	7.93	108
hotspot 2D.1	2D	8–9	P	BS00067698_51	14860348	BS00047901_51	15967448	1.11	30
hotspot 3B.1	3B	37–38	C	Tdurum_contig43252_1762	23782080	TA001028-0737	24007966	0.23	8
hotspot 3B.3	3B	67–69	C,P	Ku_c27771_508	495471559	BS00030430_51	503989868	8.52	52
hotspot 4B.1	4B	55–57	C	Ra_c26080_461	36642697	BS00095416_51	40233919	3.59	30
hotspot 5A.2	5A	84–86	C	BS00073670_51	570716220	Excalibur_c472_914	568272220	2.44	40
hotspot 5A.3	5A	90–92	P	Kukri_c10033_724	584677742	Excalibur_c26671_57	591319197	6.64	87
hotspot 5A.4	5A	115–116	C	BS00076948_51	664273096	Tdurum_contig11521_102	665779594	1.51	20
hotspot 5B.2	5B	60–61	C	RAC875_c38511_91	476805518	RAC875_c2437_1569	479025121	2.22	39
hotspot 6B.1	6B	39–40	C	RAC875_c2291_123	41705928	RAC875_c13920_836	42778537	1.07	23
hotspot 6B.2	6B	73–74	E	BobWhite_c28409_462	635175311	wsnp_Ex_c1276_2445537	642348416	7.17	39
hotspot 6B.3	6B	84–86	P	Kukri_c58961_76	669019620	Tdurum_contig68217_361	674946651	5.93	50
hotspot 6B.4	6B	120–121	P	Kukri_c60966_261	719509426	Tdurum_contig10729_989	720983865	1.47	25
hotspot 7A.3	7A	207–211	E	BobWhite_c32347_219	708145137	Ku_c19745_1093	712058458	3.91	74
hotspot 7A.4	7A	215–218	E	BS00027226_51	717965474	Kukri_c9728_1171	719567332	1.60	14
hotspot 7A.5	7A	227–229	C,E	BS00020236_51	730426125	Tdurum_contig46717_2021	731267973	0.84	18
hotspot 7B.1	7B	57–59	C,P	GENE-4826_641	61557711	BS00091302_51	64726430	3.17	31

**Table 4:** QTL hotspots involved in the differentiation patterns showing allelic differences among subpopulations.

QTL Hotspot	Marker	Position (cM)	Position (bp)	Allele (Frecuency)		
				SP1	SP2	SP3
hotspot 2B.1	Kukri_c35153_956	102.2	539,965,301	A (54%)	A (25%)	A (91%)
				G (46%)	G (75%)	G (9%)
	Kukri_c35153_145	104.4	539,964,058	A (46%)	A (75%)	A (9%)
				G (54%)	G (25%)	G (91%)
hotspot 5B.1	wsnp_BE443187B_Ta_2_1	51.2	410,531,891	A (4%)	A (25%)	A (77%)
				C (96%)	C (75%)	C (23%)
hotspot 5B.3	RAC875_c19099_434	68.9	519,153,286	T (92%)	T (67%)	T (18%)
				C (8%)	C (29%)	C (82%)
	Ra_c73292_443	69.2	513,607,799	A (8%)	A (46%)	A (55%)
				G (92%)	G (46%)	G (45%)
hotspot 6B.2	GENE-1074_108	73.4	633,906,287	T (92%)	T (4%)	T (32%)
				C (8%)	C (96%)	C (68%)
	GENE-4086_659	73.4	-	A (4%)	A (50%)	A (36%)
				C (96%)	C (50%)	C (64%)
	GENE-4086_876	73.4	641,286,640	A (4%)	A (46%)	A (36%)
				G (96%)	G (50%)	G (64%)
	Kukri_c59960_211	73.4	641,291,882	T (4%)	T (46%)	T (36%)
				C (96%)	C (50%)	C (64%)
hotspot 7A.4	Excalibur_c46453_144	217.0	719,568,282	A (19%)	A (50%)	A (59%)
				G (81%)	G (46%)	G (41%)
	Kukri_c9728_1171	217.0	719,567,232	A (81%)	A (46%)	A (41%)
				G (19%)	G (50%)	G (59%)
hotspot 7B.2	wsnp_Ex_c106_217340	76.0	538,868,953	A (96%)	A (58%)	A (100%)
				G (4%)	G (33%)	G (0%)
	Kukri_c51296_438	77.1	565,911,626	A (15%)	A (96%)	A (91%)
				G (85%)	G (4%)	G (9%)
hotspot 7B.2	wsnp_Ex_c5270_9324025	77.1	-	T (15%)	T (96%)	T (91%)
				C (85%)	C (4%)	C (9%)
hotspot 7B.2	wsnp_RFL_Contig4753_5709032	77.1	566,481,254	A (85%)	A (4%)	A (9%)
				G (15%)	G (96%)	G (91%)

**Table 5:** Summary of QTL hotspots in syntenic chromosomes.

Chromosome	Hotspot	Syntenic Chromosomes						
1A	hotspot 1A.1	bd2	os5					
1A	hotspot 1A.2	bd2	os5	zm6				
1A	hotspot 1A.4	bd2	bd3	os5				
1B	hotspot 1B.1-2	bd1	bd3	os1	os2	os5	os7	os12
1B	hotspot 1B.3	bd2						
1B	hotspot 1B.5	bd2	bd3	os1	os5	os6	os7	zm6
1B	hotspot 1B.6	bd1	bd2	os5	os6			
1B	hotspot 1B.7	bd2	os5	zm8				
1B	hotspot 1B.8	bd2	os4	os5				
1B	hotspot 1B.9	bd4	os4	os9				
1D	hotspot 1D.1-2	bd1	bd2	os5				
2A	hotspot 2A.1	os7						
2A	hotspot 2A.3	bd3	os1	os4				
2B	hotspot 2B.3	bd2	bd4	bd5	os4	os9	zm2	zm8
2D	hotspot 2D.1	bd4	os11					
3B	hotspot 3B.3	bd2	os1	os5				
5A	hotspot 5A.3	bd1	bd2	os1	os3			
5A	hotspot 5A.4	bd1	os3	zm1				
5B	hotspot 5B.2	bd2	bd4	os1	os9			
6B	hotspot 6B.1	bd3						
6B	hotspot 6B.3	bd4	bd5					
6B	hotspot 6B.4	bd3	os2	zm5				
7A	hotspot 7A.3	bd1	os2	os6				
7A	hotspot 7A.5	bd1	os2	os6	zm6			

## Data Availability

The data presented in this study are available within the article and its supplementary data, or on request to the authors.

## Supplementary Materials

The following supporting information can be downloaded at: https://www.mdpi.com/article/10.3390/ijms24021700/s1.
